# I Just Have to Try Harder: Examining the Mindsets of Students with LD

**DOI:** 10.1177/0829573521998954

**Published:** 2021-03-04

**Authors:** Lauren D. Goegan, Gabrielle N. Pelletier, Lia M. Daniels

**Affiliations:** 1 University of Alberta, Edmonton, Canada

**Keywords:** learning disability, exceptionalities/disabilities, motivation, personality/individual differences, achievement, secondary education/adolescence

## Abstract

Growth and fixed mindset messaging is gaining popularity. In our pilot study, we examine the mindsets of students with learning disabilities (LD) to determine how their self-beliefs relate to this messaging. Our results demonstrate that students with LD endorse growth mindsets more than fixed mindsets which is consistent with their peers without LD. Moreover, in their comments about being a student with LD, participants highlight important components of growth mindset messaging. However, some comments may reflect a false-growth mindset wherein students are only focused on effort and not the additional resources required for growth. We provide directions for future research.

Students with learning disabilities (LD) can experience a variety of challenges within the classroom. According to the Diagnostic and Statistical Manual of Mental Disorders (DSM-5, American Psychiatric Association, 2013), a specific learning disorder involves difficulties learning and using academic skills (p. 66). As a result of the challenges experienced by these individuals, educational supports are often required during their academic pursuits. This can involve the implementation of appropriate accommodations such as extended time (Goegan & Harrison, 2017) or the implementation of assistive technology (Goegan et al., 2019). These supports are often executed based on the recommendations of school psychologists articulated in psychoeducational reports. Another avenue for supporting students with LD could be to examine their self-beliefs about being a student with LD. For the purposes of this paper, self-beliefs are one’s views about themselves that shape their experiences and actions (Dweck, 2008).

## Mindset Theory

We draw on Dweck’s (1999) mindset theory as the guiding framework for this pilot study. Based on this theory, students’ beliefs about intelligence and other attributes align with either a growth or a fixed mindset. Students with a growth mindset believe that intelligence or abilities are malleable, and can change with effort or applicable learning strategies. For example, a student may think “*If I study hard, I can do well on the exam in my class*.” Students with a fixed mindset believe that intelligence is innate and therefore permanent and unchanging (Yeager & Dweck, 2012). For example, a student may think “*I will never do well on the exam in my class because it’s just not my thing.*” Over the years, researchers have identified patterns in the experiences and actions among students based on these beliefs. For example, students who hold a fixed mindset have been found to experience frustration and lower performance on, or even withdrawal from, challenging tasks; whereas, students with a growth mindset put in more effort, view challenging tasks as an opportunity for improvement and achieve higher grades (Dweck & Yeager, 2019). Not surprisingly, growth mindsets are considered more adaptive relative to fixed mindsets (e.g., Blackwell et al., 2007; Dweck & Yeager, 2019). As such, fostering a growth mindset may be an important avenue to supporting students with LD in their academic pursuits.

### Mindsets in students with LD

There have been some researchers that have examined mindsets and students with LD. Tuckwiller et al. (2017) surveyed students with LD from grades 11 and 12 and determined they were more likely to endorsed a growth mindset than a fixed mindset. Researchers have also examined students with LD in comparison to their peers. Baird et al. (2009) examined students with LD in grades 6 through 12 to assess their self-beliefs, including mindsets. Their results suggested that compared to non-LD students, students with LD were more likely to believe that intelligence was fixed. More recently, there have been mixed findings as some researchers found that students with LD tend to endorse a growth mindset more than their peers (e.g., Matheson, 2015), while others found no difference in mindset beliefs (e.g., Blake, 2015). Therefore, we were interested in examining mindsets within a group of students with LD and relative to their peers to bring additional evidence to bear on the endorsement of mindsets by students with LD.

### False growth mindsets

Mindset theory has grown in popularity over the decades. A recent search on Google Scholar found Dweck’s book, *Mindsets: The New Psychology of Success*, was cited over 10,000 times. As access to ideas and materials related to mindsets have become almost commonplace, a challenge has emerged as consumers may unintentionally misunderstand or misuse the theory leading to what is known as a false growth mindset. False growth mindsets occur when individuals focus solely on the effort component of a growth mindset while overlooking the connection between effort and other important educational considerations like strategy enactment or mentorship (Dweck & Yeager, 2019). For example, when a student with LD struggles with reading comprehension, growth mindset needs to be enacted by focusing on effort and also equipping students with appropriate instruction and strategies that correspond to their learning needs.

Increasing demands of higher grade-levels and postsecondary education may result in students with LD requiring further effort and additional effective academic strategies. An increased need for effort required by students with LD may also lead to increased fatigue. Ben-Naim et al. (2017) found that college students with LD report higher levels of tiredness as compared to their non-LD peers. Moreover, researchers have also found that feelings of fatigue correspond to reduced academic performance (Mizuno et al., 2011) and cognitive functioning (Palmer, 2013). Therefore, promoting a growth mindset while seemingly positive, could result in negative outcomes for students with LD if they increase their effort without appropriate strategies. In this study, we were interested in examining students in grade 12, as this is an important transition year for many students in the province in which this study was conducted. Many of them intend to pursue postsecondary education after graduation which requires additional effort and therefore, represents a particularly vulnerable time wherein a false growth mindset may be particularly detrimental. As such, we were interested in examining mindset messaging, in particular, false growth mindsets and the self-beliefs of students with LD to make a contribution to this emerging literature from the perspective of students with LD.

## The Current Study

In the current pilot study, we explored the mindsets of grade 12 students with LD from one midsized Canadian province in Western Canada. Understanding their mindsets is important because currently there are mixed findings as to whether these students endorse a growth or fixed mindset. Moreover, by asking students with LD about how their self-beliefs about having a LD, we hope to add to the emerging literature on false growth mindsets. For the students surveyed, grade 12 is an important academic year as many will be transitioning to postsecondary education which requires increased academic demands necessitating increased effort and effective strategies—consistent with a growth mindset. Therefore, our research questions were as follows:

1. Do students with LD score similar to peers on measures of fixed and growth mindsets?
2. Within the group, do students with LD identify more with a growth or fixed mindsets?
3. How do students’ self-beliefs about having a LD correspond with mindset messaging?

## Methods

### Participants and Procedures

Data were collected from 100 grade 12 students from several high schools in a western Canadian province. Half of the students self-identified as LD (*n* = 50) and the other half were randomly selected from a larger sample of students who did not report having a LD. Self-identification as a student with LD was determined by students’ response to the question “Do you have a learning disability?” We utilized self-identification for LD as psychoeducational assessments were not available to the researchers and self-reporting methods have been suggested to be an effective and ecologically valid way for determining individuals with LD in research (e.g., McGonnell et al., 2007).

The LD sample consisted of 27 males, 20 females, and three students who did not identify their gender. The sample of non-LD students consisted of sample consisted of 25 males, 24 females, and one student who did not identify their gender. The average participant age was 17.4 years old (*SD* = .68). Information was sent home to parents informing them of the study, and the opportunity to contact the researchers for more information. The survey was completed at home, or school depending on the guidelines of the individual schools. This study was approved by the University’s Research Ethics Board.

### Measures

#### Mindset measure

Participants completed the theories of intelligence scale developed by Dweck (1999). All eight items were completed on a response scale from 1 (strongly disagree) to 6 (strongly agree). Four of the items were summed to create a fixed mindset total score (e.g., you have a certain amount of intelligence, and you can’t really do much to change it; α = .86) and the remaining four items were summed to create a growth mindset total score (e.g., you can always substantially change how intelligent you are; α = .92).

#### Self-beliefs

Participants who self-identified as having a LD provided written responses to the open-end prompt “What does it mean TO YOU to have a learning disability?”

### Plan for Analyses

Two independent samples *t*-tests were performed to examine our first research question, that is, do students with LD score similar to their peers on measures of fixed and growth mindsets. A Bonferroni correction was applied across all *t*-tests to control for Type 1 error (*p* < .017). A paired samples *t*-test was performed to examine our second research question. Finally, we utilized a summative approach to qualitative content analysis, by first entering the responses that students provide into a word cloud software to identify and quantify the words that could be classified as growth or fixed (Hsieh & Shannon, 2005). Use of word cloud software has recently been used by other researchers to examine participants’ open-ended responses and the most common words utilized (e.g., Hinkle et al., 2020; Meehan & Howells, 2019). We utilize the word cloud software here to examine the words utilized by students who self-identify as having an LD to make connections between participants’ open-ended responses and mindset messaging.

## Results

Students with LD identified a fixed mindset similar to their peers *t*(99) = .44, *p* = .662 (*M* = 12.04, *SD* = 4.51 and *M* = 11.64, *SD* = 4.63 respectively), as well as a similar growth mindset *t*(99) = .51, *p* = .608 (*M* = 17.02, *SD* = 4.61 and *M* = 17.48, *SD* = 4.29 respectively). Therefore, students with LD and their peers have similar levels of growth and fixed mindsets. When compared within the group, students with LD reported significantly higher growth than fixed mindsets scores, *t*(49) = 4.34, *p* < .001, *d* = .59.

**Figure 1.**
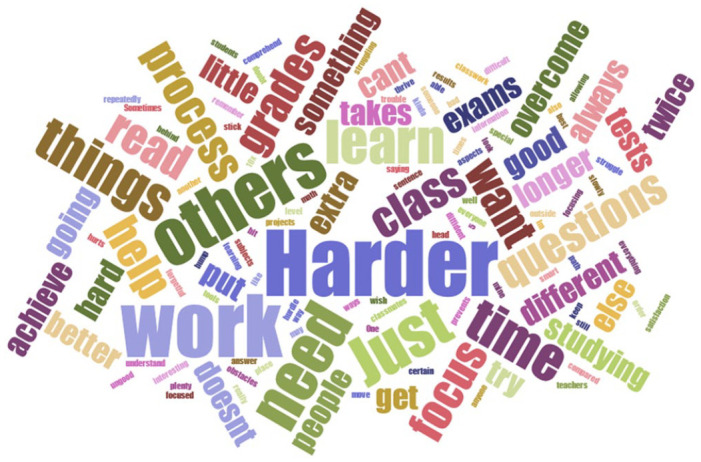
Word cloud—what does it mean to you to have a learning disability?

The results of the word cloud, after removing high-frequency words such as “the” or “and” and words found in the question itself like “means” and “LD,” are presented in Figure 1. In the figure, word size is related to frequency with larger words having been used more frequently in students’ responses. To examine word usage, we put the most common words utilized by participants in Table 1 along with representative quotes. Moreover, Table 1 also includes target words in bold with other high frequency words identified in gray/bold, to examine how the words “cover the data” as a form of enhancing the trustworthiness and credibility of the interpretation (Elo et al., 2014, p. 7). Next, we use representative quotes (Elo et al., 2014; Hsieh & Shannon, 2005) to describe the word cloud results and their link to growth or fixed mindsets.

**Table 1.** Most Common Words Utilized in Response to: What Does it Mean to You to Have a Learning Disability?

Word	Response rate (%)	Example response
Hard/Harder	31	That I have to look and try a bit **harder** but it doesn’t put me in a different place than **others**.
Work	25	One bump in my path that I **just** have to **work harder**.
Others	18	**Things** are **harder** for me than **others**.
Need	16	There are certain aspects of **learn**ing that I may behind and **need** special tools to **help** me be on the same level as **others**.
Learn	13	I **learn** a different way.
Things	13	I have trouble **focus**ing and plenty of other **things**.
Time	13	To have a learning disability you **need** to **learn** in different ways than **others** do or **need** more **time** to **process** information.
Class	9	I cannot **focus** as well in **class** and I have to **work** twice as **hard** as my classmates (and studying takes twice the **time** it should).
Focus	9	I can’t **focus**. I have to **read** it five **times** over before I can **process** what it’s saying.
Grades	9	I have to **work** extra **hard** to get good **grades**.
Help	9	That I **need** a little more extra **help** then anyone else.
Just	9	I **just** have to try **harder**.
Process	9	That it takes me longer to **process** and answer **questions**.
Questions	9	To not understand the **questions** because you can’t remember them.
Read	9	I **read** a sentence and it doesn’t stick.
Want	9	In my **class just** I have to **work harder** for what I **want**.

*Note*. Target word identified in bold, other high frequency words identified in gray/bold.

Harder was the most frequently utilized word by students and was extracted from comments such as, “I just have to try *harder*,” and “I have to work *harder* for what I want.” Overall, students’ use of the word “harder” implied a high level of effort, which is consistent with a growth mindset. Students also made several references to work. For example, “I have to *work* extra hard to get good grades,” and “I will always have to *work* 10x harder than them.” These comments also reflected the effort component of a growth mindset, while acknowledging that they view their level of effort as more than their peers.

The comparison to others was often presented in their responses. This can be seen in the response “you need to learn in different ways than *others.*” In their responses, students often commented on what they need, for example, “you *need* to learn in different ways than others do” or “*need* more time to process information.” Needs can also be seen in the comments related to help, for example “I need a little more extra *help*” Moreover, these needs were often described in relation to time, for example, “I *need* longer on projects and exams”. Time was also described as a requirement to complete tasks, for example, “studying takes twice the *time.*” In general, students’ comments were contextualized largely within the school setting as evidence by high use of words such as “class,” “grades,” “read,” “questions,” and “learn”. Students’ comments made in comparison to others and concerning their learning needs at school might speak to the strategies and instruction required in addition to the effort component of a growth mindset. Overall, the main words matched better with the notion of growth than fixed mindsets.

However, there was one word that is an exception and that is the word *just.* Indeed, many used the term to qualify their experiences such as “I *just* have to try harder,” “one bump in my path that I *just* have to work harder,” and “*just* another hurdle to overcome.” These comments may suggest an optimism that additional effort to their work is all that is required. Indeed, this optimism may suggest a false growth mindset, as these students are focused more on the effort required rather than the full cost of the commitment, such as necessary supports, or acknowledging the fatigue that can occur with increased effort.

## Discussion

Our research examined the endorsement of mindsets by students who identified as having an LD in grade 12. In this discussion, we focus on how these findings expand our current understanding of self-beliefs of students who identify with having a LD and potential avenues for further investigation. Specifically, we discuss how these students do not differ from their peers in levels of mindsets. Also, students who identify with having a LD appear more growth- than fixed-oriented in their mindsets in terms of scores and descriptions; however, we offer a caution regarding false mindsets. Following this, we offer suggestions for future research and recommendations for school personnel.

### Students with LD Do not Differ from Their Peers in Mindsets

Based on our results, students with LD identified similar levels of growth and fixed mindsets when compared to their peers. While previous research is mixed on this finding (e.g., Blake, 2015; Matheson, 2015), we are optimistic that students, regardless of disability status are endorsing a growth mindset in our sample. Indeed, teachers should be providing messaging to all their students that they can indeed grow with effort and appropriate implementation of learning strategies and supports (Dweck, 2007). The positive behaviors associated with a growth mindset, such as taking on challenges and working hard, should be fostered in all students throughout their schooling (Dweck, 2007). Endorsement of a growth mindset is particularly important for students who are transitioning to postsecondary education as they will be required to navigate new academic demands and challenges, that require the implementation of effort and learning strategies previously acquired.

### Endorsement of a Growth Mindsets

From a within group perspective, students who identified with having a LD endorsed a growth mindset more than a fixed mindset. These students scored more than one point higher on average when it came to growth mindset items. This is important given the positive behaviors associated with having a growth mindset as mentioned above (Blackwell et al., 2007; Dweck & Yeager, 2019). For students with LD in particular, who may experience additional difficulties with learning (American Psychiatric Association, 2013), a belief that effort will enhance ability, or a desire to take on challenges could be especially beneficial.

Moreover, the open-ended comments from students who identify with having a LD also demonstrated a growth mindset. Many students’ comments related to effort, which is a key component of mindset messaging. Furthermore, students commented on what they need to be successful (e.g., needing special tools), such as assistive technology, or other accommodations. In particular, time was mentioned regularly. Overall, these comments suggest a growth mindset, such as the need for effort to be successful, in addition to effective instruction and learning strategies to utilize their effort effectively.

Nevertheless, statements concerning effort and having to work “harder” were more frequent than comments related to strategies which may suggest some students may have a false growth mindset. Moreover, the use of the word “just” may also reflect a false growth mindset. This can be problematic if students are simply equating their challenges with trying harder and not the strategies and supports that come alongside those challenges. This is consistent with the notion of a false-growth mindset that suggests that individuals with such a mindset do not fully grasp the concept and simply equate effort with growth (Dweck & Yeager, 2019). Then again, the use of the word *just* could imply some optimism students have about their abilities as a student with LD. However, caution is needed as a literature review by Klassen (2002) found that students with LD appear to be overly optimistic about their abilities.

While students with LD mention additional effort required, they did not mention associated fatigue or other emotions. Research has found that students with LD reported higher levels of tiredness compared to their non-LD peers (Ben-Naim et al., 2017), and increased fatigue is associated with a decrease in cognitive functioning and academic performance (Mizuno et al., 2011; Palmer, 2013). This reflects an area of needed investigation because if students are reporting that their LD means “just having to try harder” this may not accurately reflect their learning needs that accompany the effort required. As a result, students may be putting in ineffective effort that might come at the cost of additional fatigue. A longitudinal research design could be a possible avenue for investigating mindsets overtime and associated outcomes like fatigue and academic emotions.

The results of our pilot study provide some preliminary information about the mindsets of self-identified students with LD in grade 12 and how their self-beliefs connect with mindset messaging. Nevertheless, there are a few limitations that should be noted. First, participants were a homogenous group of students from one province in Western Canada. Future research could extend our research to other regions. Second, while we utilized self-report data to examine self-beliefs of students who identify as having an LD, an examination of psychoeducational assessments and students’ academic history could provide important additional information such as whether there are discrepancies between types of LD or whether their self-beliefs align with their diagnosis.

Various future directions could be taken with our research moving forward. For example, students who identify as having a LD could be interviewed about their mindsets, and outcomes such as fatigue and academic success to extend the knowledge obtained in this study. In particular, enquiring when students utilize the word ‘just’ or ‘hard’ when discussing their LD could provide information about potential false growth mindsets. Moreover, future research could be conducted to examine the communication of mindsets messaging from teachers and other school personnel and how the information is adopted by students generally, and students who identify with having a LD in particular, to support the development of accurate growth mindsets. Our findings may be particularly important for school psychologists when discussing a potential LD diagnosis to ensure effort is not overemphasized. Rather, the combination of effort and effective learning strategies and supports should be encouraged. By better understanding the mindsets of students with LD, researchers, administrators, teachers, and other stakeholders are better positioned to move forward with appropriate and meaningful supports.
